# Acute kidney injury is a powerful independent predictor of mortality in critically ill patients: a multicenter prospective cohort study from Kinshasa, the Democratic Republic of Congo

**DOI:** 10.1186/s12882-016-0333-4

**Published:** 2016-08-24

**Authors:** Angèle Masewu, Jean-Robert Makulo, François Lepira, Eric Bibonge Amisi, Ernest Kiswaya Sumaili, Justine Bukabau, Vieux Mokoli, Augustin Longo, Yannick Nlandu, Yannick Engole, Cedric Ilunga, Alphonse Mosolo, Alex Ngalala, Justin Kazadi, Richard Mvuala, Jackson Athombo, Nkodila Aliocha, Pierre Zalagile Akilimali, Adolphe Kilembe, Nazaire Nseka, Michel Jadoul

**Affiliations:** 1 Intensive Care Unit, Faculty of Medicine, University of Kinshasa Hospital, University of Kinshasa, Kinshasa, Democratic Republic of Congo; 2 Nephrology unit, Department of internal medicine, Faculty of Medicine, University of Kinshasa Hospital, University of Kinshasa, Kinshasa, Democratic Republic of Congo; 3 Intensive Care Unit, Centre Hospitalier de Monkole, Kinshasa, Democratic Republic of Congo; 4 Intensive Care Unit, Hôpital Sino-Congolais de Kinshasa, Kinshasa, Democratic Republic of Congo; 5 Intensive Care Unit, Centre Médical de Kinshasa, Kinshasa, Democratic Republic of Congo; 6 Intensive Care Unit, Hôpital Général Provincial de Référence de Kinshasa, Kinshasa, Democratic Republic of Congo; 7 Intensive care unit, Clinique Ngaliema, Kinshasa, Democratic Republic of Congo; 8 Intensive care unit, Hôpital Biamba Marie Mutombo, Kinshasa, Democratic Republic of Congo; 9 Kinshasa School of Public Health, University of Kinshasa, Kinshasa, Democratic Republic of Congo; 10 Division of Nephrology, Cliniques Universitaires Saint-Luc, Université Catholique de Louvain, Brussels, Belgium

**Keywords:** Acute kidney injury, Intensive care unit, Incidence, Mortality, Black Africans

## Abstract

**Background:**

Despite the growing incidence of acute kidney injury (AKI) worldwide, there is little data on the burden and outcomes of AKI in intensive care unit (ICU) in low resource settings. The present study assessed the incidence of AKI and its impact on mortality in ICU in Kinshasa (Democratic Republic of Congo).

**Methods:**

In a prospective cohort study, 476 consecutive critically ill patients (mean age 52 years, 57 % male) were screened for the presence of AKI in seven ICU from January 1st to March 30th, 2015. Serum creatinine was measured by the enzymatic method (Cobas C111 device®). AKI and its stages (no AKI, AKI 1, AKI 2 and AKI 3) were defined according to AKIN recommendations. The primary outcome was 28 days mortality. Survival (time-to death) curves were built using the Kaplan Meier methods. Predictors of mortality were assessed by Cox proportional hazards regression models. *p* < 0.05 defined the level of statistical significance.

**Results:**

The cumulative incidence of AKI was 52.7 % with AKI stage 1, 2 and 3 in 23.7 %, 16.2 % and 12.8 % of patients, respectively. Among patients who developed AKI, 146 died (58 %) vs 62 patients (28 %) in the group without AKI. Only 6.5 % of the patients with AKI stage 3 benefited from dialysis. Median survival time was 15.0 days in patients without AKI and 3.0 days, 6.0 days and 8.0 days in patients with AKI stage 3, 2 and 1 (*p* < 0.001), respectively. In addition to respiratory distress-induced polypnea (HRa 1.60; 95 % CI: 1.08–2.37; *p* = 0.018), oxygen desaturation (HRa 1.53; 95 % CI: 1.13–2.08; *p* = 0.006) and multi-organic involvement (HRa 1.63; 95 % CI: 1.15–2.30), AKI emerged as an independent predictor of death (HRa 1.82; 95 % CI: 1.34–2.48; *p* < 0.001).

**Conclusion:**

More than half of critically ill patients in the present cohort developed AKI which contributed substantially to short-term mortality, highlighting the need for its prevention, early detection and management as well as the availability of dialysis in ICU.

## Background

Acute kidney injury (AKI) is an abrupt loss of kidney function which occurs within a few hours or days. It may lead to a number of complications like high serum potassium levels, metabolic acidosis, imbalance in body fluid, uremia and finally death [1]. The new AKI terminology of acute renal failure stresses that the disease spectrum extends from less severe forms of injury to more advanced injury when acute kidney failure may require dialysis [1].

AKI is especially common in hospitalized patients, particularly in critically ill patients who need intensive care. Its incidence is growing worldwide because of the aging population and associated multiple comorbidities [2, 3]. The systematic measurement of serum creatinine and quantification of hourly diuresis favor the comprehensive detection of AKI in hospitalized patients.

In Western countries, the frequency of AKI in ICU has been reported to vary from 5% to 60 % [4, 5]. The lack of standardization of the definition of AKI until recently can explain the differences between studies. AKI in ICU is associated with high mortality, longer hospital stay and greater costs especially in patients requiring hemodialysis [6]. In sub-Saharan Africa (SSA), the situation is even more crucial since most ICU are poorly equipped and staffed [7]. Indeed, AKI is a challenging problem in low-resource settings because of the high burden of infectious diseases such as HIV and malaria, the over the counter availability of potentially nephrotoxic drugs and medicinal plants, the late presentation of patients to healthcare services and the lack of hemodialysis centers which, when present, are not financially accessible to the vast majority of the population [8, 9]. Despite the ever growing incidence of AKI worldwide, there is little data on the burden and outcomes of AKI in ICU in low resource settings.

In the Democratic Republic of the Congo (DRC), acute renal failure, prior to the standardization of AKI definition, has been reported to be a common finding in hospitalized patients [9]. We therefore took advantage of the standardization of AKI definition to generate data on its burden in ICU. The aim of the present study was thus to determine the overall incidence and outcomes of AKI in ICU in Kinshasa/DR Congo.

## Methods

The study was conducted in seven ICU: University hospital of Kinshasa, Clinique Ngaliema, Centre hospitalier Monkole, Hôpital Biamba Marie Mutombo, Centre médical de Kinshasa, Hôpital général provincial de référence de Kinshasa, hôpital de l’amitié sino-congolaise from January1^st^ to March 30th , 2015. All patients aged ≥20 years, hospitalized during the study period were included in the study. A survey form was used to collect demographic information (gender, age), the patient’s medical history (hypertension, diabetes mellitus, stroke, cancer, heart failure, chronic kidney disease, last serum creatinine measurement, HIV positivity, cirrhosis, obstructive uropathy, sickle cell anemia), lifestyle habits (alcohol intake, smoking, NSAIDs and medicinal plants intake), the cause of ICU admission, the number of vital organs with dysfunction, peripheral oxygen saturation and diuresis. Comorbidities were classified according to the International Classification of Diseases-10 (ICD-10). Serum creatinine was measured using the enzymatic method (Cobas C111 device®). The highest value of serum creatinine was used to stage AKI using the Acute Kidney Injury Network (AKIN) with different stages of AKI (no AKI, AKI 1, AKI 2 and AKI 3) [1]. Data related to the outcome of AKI were the duration of hospitalization, survival (time-to-death), hemodialysis start or not and the vital status until the 28th day.

Statistical analyses were performed using SPSS 21.0 for Windows (SPSS Inc., Chicago, IL, USA). Comparisons between groups were performed using Student’s t test, Fisher’s exact test, Mann-Whitney test and Chi square test, as appropriate. Kaplan Meier curves were built for survival analyses. Cox regression analysis was used to identify independent predictors of mortality. Association measures were calculated with 95 % confidence intervals. *P* < 0.05 defined the level of statistical significance.

The study protocol was approved by the ethical committee of the School of public health of the University of Kinshasa. Written informed consent was obtained from the study participants. For patients unable to give consent because of severity of illness, the next of kin was identified and gave informed consent.

## Results

### General characteristics of patients hospitalized in ICU

The study population consisted of 476 patients, 269 men and 207 women. Patients with medical conditions numbered 393 (82.6%) and those with surgical conditions 83 (17.4%). The age of patients was 51.9 ± 17.9 (range 20 to 99) years with a significant difference between genders (men: 53.4 ± 16.9 years vs women: 49.9 ± 18.9 years; *p* = 0.036). More than half of patients (74.4%) came from other hospitals while 25.6 % were transferred from the emergency or other units of the same hospital because of worsening status.

### Overall incidence and causes of AKI

The overall incidence of AKI was 52.7 % with AKI stage 1, 2 and 3 in 23.7 % 16.2 % and 12.8 % of patients, respectively. Table 1 shows that patients with AKI were taking NSAIDs more frequently and were more likely to have CKD compared with the group without AKI. Hypertension and diabetes were the most common comorbidities, whatever the group studied (AKI vs no AKI). Patients who had AKI stage 2 or 3 were older than patients without AKI or with AKI stage 1.

**Table 1:** Characteristics of hospitalized patients with AKI vs no AKI

Characteristics	no AKI (*n* = 225)	AKI 1 (*n* = 113)	AKI 2 (*n* = 77)	AKI 3 (*n* = 61)	*p* value
Age	50.6 ± 17.2	49.9 ± 17.8	56.7 ± 19.1	54.9 ± 18.0	0.020
Men	122(54.2)	67(59.3)	40(51.9)	40(65.6)	0.320
K > 5.5 mmol/l HC03- < 15 mmol/l	5(3.2)^a^ 1(1.1)^a^	10(12.7)^a^ 16(29.6)^a^	9(18.8) ^a^ 11(29.7)^a^	21(44,6)^a^ 16(57.1)^a^	0.001 0.001
GCS < 9	28(12.4)^a^	20(17.6)^a^	15(19.7)^a^	9(14.7)^a^	0.029
Hypertension	83(37.1)	52(46.0)	23(29.9)	26(42.6)	0.127
Diabetes	30(13.4)	23(20.4)	7(9.1)	10(16.4)	0.158
Stroke	18(8.0)	10(8.8)	7(9.1)	6(9.8)	0.945
Cancer	16(7.1)	10(8.8)	4(5.2)	5(8.2)	0.794
Congestive heart failure	12(5.4)	2(1.8)	7(9.1)	6(9.8)	0.056
HIV/AIDS	8(3.6)	2(1.8)	2(2.6)	2(3.3)	0.833
NSAIDs	7(3.1)	10(8.8)	4(5.2)	4(6.6)	0.013
CKD	4(1.8)	10(8.8)	3(3.9)	3(4.9)	0.022

Values are n (%) or means ± SD

*Abbreviations*: *HIV/AIDS* Human immunodeficiency virus infection and acquired immune deficiency syndrome, *NSAIDs* non-steroidal anti-inflammatory drugs, *CKD* chronic kidney disease, *GCS* Glasgow coma scale

^a^Results not available in all patients in the study

The causes of AKI are detailed in Table 2. According to ICD-10, infectious diseases and cardiovascular system diseases tended to be associated with AKI, but without significant difference compared to the group without AKI. Metabolic, genitourinary system and digestive system diseases were more frequent in the group with AKI (Table 2).

**Table 2:** Association between diseases groups according to ICD-10 and AKI

Diseases groups	no AKI (*n* = 225)	AKI 1 (*n* = 113)	AKI 2 (*n* = 77)	AKI 3 (*n* = 61)	*p* value
Infectious and parasitic	104(46.2)	66(58.4)	36(46.8)	34(55.7)	0.135
Circulatory system	104(46.2)	48(42.5)	29(37.7)	26(42.6)	0.616
Endocrine, nutritional and metabolic	75(33.3)	56(49.6)	28(36.4)	21(34.4)	0.033
Genitourinary system	44(19.6)	33(29.2)	21(27.3)	17(27.9)	0.048
Respiratory system	43(19.1)	23(20.4)	9(11.7)	13(21.3)	0.370
Digestive system	22(9.8)	14(12.4)	11(14.3)	13(21.3)	0.020
Blood, blood forming organs and immune mechanisms	17(7.6)	15(13.3)	10(13.0)	5(8.2)	0.265

*Abbreviations*: *ICD* international classification of diseases

**Table 3:** Risk factors and all-cause mortality in critically ill patients

Characteristics	n	PD	Deaths	Mortality rate	Hazard ratio			
				(per 1000 PD) ^b^	Crude (95%CI)	*p*	Adjusted ^a^ (95%CI)	*p*
AKI								
- no AKI	225	1420	62	43.7	1		1	
- AKI	251	1451	146	100.6	2.30 (1.71–3.10)	<0.001	1.82 (1.34–2.48)	<0.001
Systems affected								
- <2	202	1066	47	44.1	1		1	
- ≥2	274	1805	161	89.2	2.32 (1.68–3.22)	<0.001	1.63 (1.15–2.30)	0.006
Peripheral oxygene saturation								
- ≥90	364	2195	131	59.7	1		1	
- <90 %	112	676	77	113.9	2.22 (1.67–2.95)	<0.001	1.53 (1.13–2.08)	0.006
Polypnea								
- <24 cycles/min	158	863	34	39.4	1		1	
- ≥24 cycles/min	318	2008	174	86.7	2.43 (1.68–3.51)	<0.001	1.60 (1.08–2.37)	0.018
Overall	476	2871	208	72.4				

Patients contributed a total of 2871 days of follow-up. Overall the mortality rate was 72.4 (95%CI: 62.6–82.3) patients per 1000 patient-days

^a^Four variables listed in the table were introduced in the Cox model

^b^PD: patient-days

### Survival estimates and predictors of mortality in critically ill patients

**Fig. 1:**
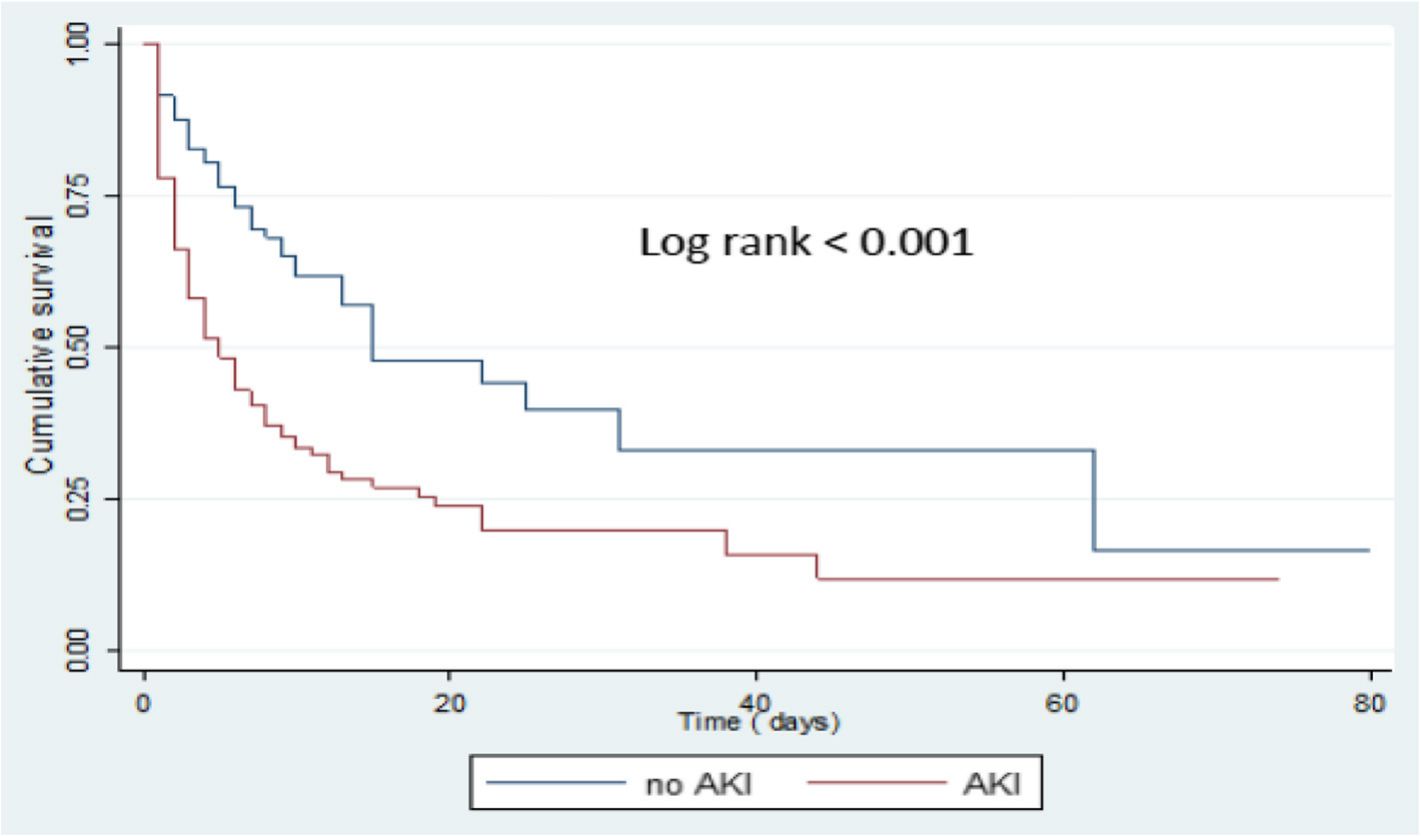
Survival in patients with AKI versus without AKI

**Fig. 2:**
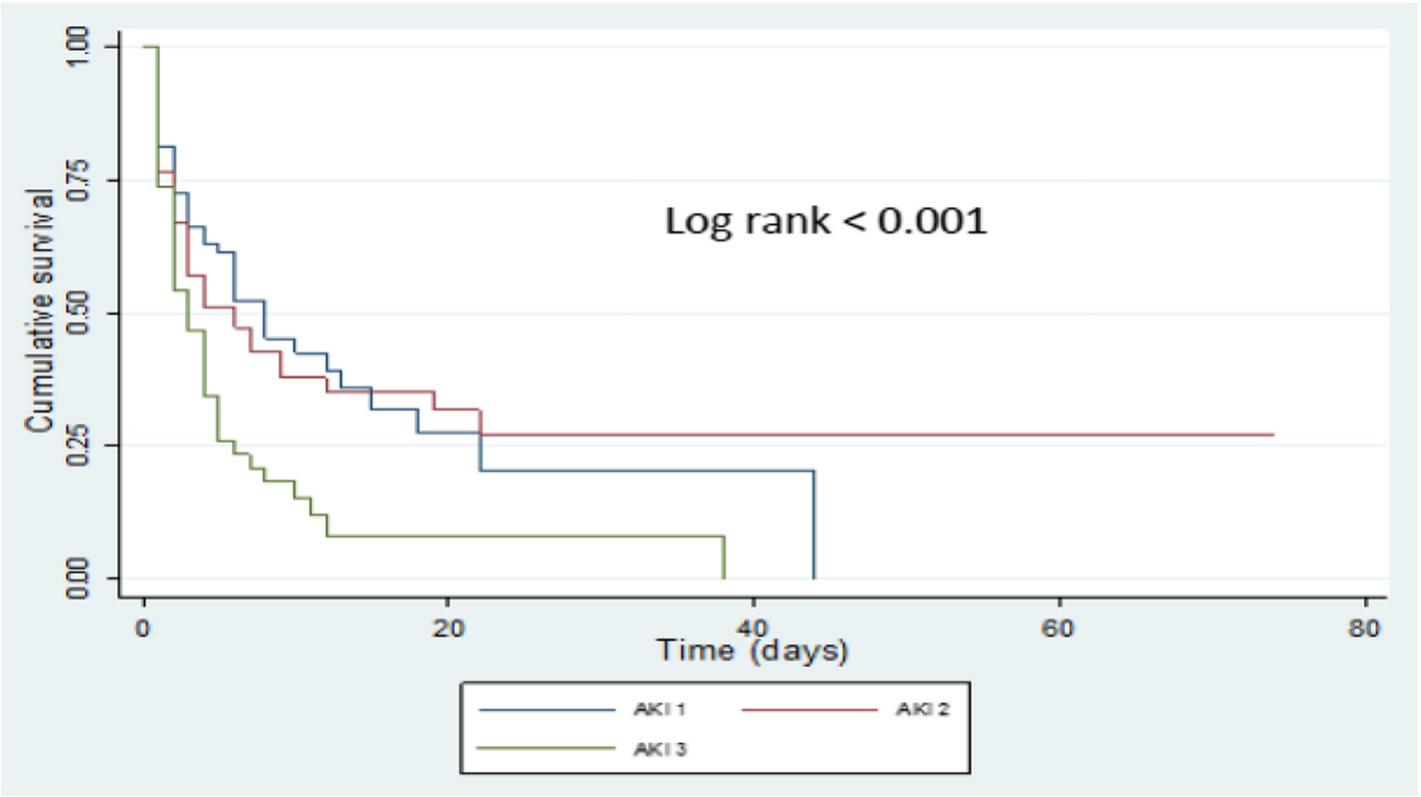
Survival in patients with AKI stage 1, 2 and 3

Among all patients with AKI stage 3, only 4 patients (6.5 %) underwent hemodialysis. Among patients who developed AKI, 146 died (58 %) vs 62 patients (28 %) in the group without AKI. The median survival time was 15.0 ± 4.7 days in patients without AKI and declined with the severity of AKI being of 3.0 ± 0.5 days, 6.0 ± 1.7 days and 8.0 ± 1.7 days in patients with AKI 3, 2 and 1 (*p* < 0.001), respectively (Figs. 1 and 2). In addition to respiratory distress-induced polypnea (aHR 1.60; 95% CI 1.08–2.37; *p* =0.018) and oxygen desaturation (aHR 1.53; 95% CI 1.13–2.08; *p* = 0.006), AKI emerged as an independent predictor of death (aHR 1.82; 95% CI 1.34–2.48; *p* < 0.001) (Table 3). The involvement of at least two vital organs or systems (aHR 1.63; 95% CI 1.15–2.30) conferred a 2-fold increase in the risk of death. The risk of dying increased significantly with the severity of AKI (Table 4).

**Table 4:** Risk of dying in patients with vs patients without AKI

Stages of AKI	n (%) of deaths	Risk vs no AKI	CI 95 %
no AKI	62 (27.5)	-	-
AKI 1	53 (46.9)	1.702	1.275–2.272
AKI 2	45 (58.4)	2.120	1.597–2.816
AKI 3	48 (78.7)	2.856	2.227–3.662

## Discussion

This prospective cohort study showed that AKI is common in critically ill patients admitted to ICU in Kinshasa (RDC) and is associated with high short-term mortality. Medical conditions, mainly infectious and cardiovascular, were the main causes of AKI, with history of CKD and NSAIDs use as main risk factors. Few patients at AKI stage 3 had access to hemodialysis and respiratory distress increased the risk of death.

The incidence of AKI in ICU (52.7 %) observed in the present study is similar to that reported in the Western World [3–5, 10–13]. Comparison of our results with other studies from SSA is very hard since most of them used non-standardized definitions of AKI [14, 15]. Previous studies acknowledged that the epidemiology of AKI in developing countries, characterized mainly by its onset at younger age and the high incidence of infectious diseases differs from that of the western world where aging and associated degenerative diseases prevail [16, 17]. However, the average age of patients of 51.9 years in the present study underlines the ongoing demographic transition experienced by SSA countries [18].

Age increased in parallel with the severity of AKI in the present study. This finding highlights the aging-induced renal morphological and functional changes that render this organ more vulnerable to AKI. Early diagnosis and prevention of AKI in these patients is vital. Patients with AKI were predominantly males. This finding agrees with previous data and can be explained by the deleterious effects of male hormones on the kidneys through accelerated apoptosis. Indeed, experimental studies of the occlusion of the renal artery demonstrated the protective role of estradiol against ischemia in female animals [19].

Although less commonly found in the present study, a history of CKD was a risk factor for AKI. It is known that AKI associated with critical illness is likely modified by the presence of CKD [20]. Similarly, the development of AKI in patients with CKD may modify the natural history of their illness and accelerate progression towards end-stage kidney disease [21]. NSAIDs intake was also significantly associated with AKI. NSAIDs can cause two different forms of AKI: haemodynamically mediated (eg, pre-renal injury and/or acute tubular necrosis) and immune mediated (eg, acute interstitial nephritis) [22]. NSAIDs reversibly inhibit the production of renal prostaglandins via the inhibition of cyclooxygenase 1 (COX-1) and COX-2. It is unclear how NSAIDs induce acute interstitial nephritis. However, it has been suggested that COX inhibition causes preferential conversion of arachidonic acid to leukotrienes, which may then activate helper T cells [22].

The most common etiologies of AKI found were infections, circulatory system (shock), metabolic and genitourinary diseases. The finding of infections as one of the main causes of AKI in the present study is consistent with the results previous reports that have found AKI a challenging problem in sub-Saharan Africa because of increased prevalence of malaria, sepsis, diarrheal disease, human immunodeficiency virus (HIV) and nephrotoxins [9, 23]. Shock whatever its origin as well as genitourinary diseases are well-known risk factors of AKI in the ICU [3–6]. The contribution of metabolic causes (largely dominated by diabetic hyperosmolar and acido-ketosis coma) is of utmost importance since it does translate the deleterious impact on the kidney of the epidemiological transition characterized by the dual epidemic of infectious and non-communicable diseases in sub-Saharan Africa [24]. In the present study, there was few cases of AKI-related to the use of potential nephrotoxic drugs (NSAIDs) and non-secured medicinal plants probably because toxicological investigations were not performed systematically.

Our analysis of a cohort of 476 patients from 7 ICU in Kinshasa using the AKIN classification showed a significant increase in the risk for mortality in patients who developed AKI compared with patients who did not. The increased risk was found to be proportional to the stage of AKI. These results are consistent with previous western studies [25]. The increased mortality rate in AKI 1 and AKI 2 poses the problem of ICU in SSA. It is about the equipment (respirator), the availability of drugs and training of intensivists without forgetting the management of late referall patients, who very often spend too long in small centers or with traditional practitioners before being transferred to hospitals [7].

For patients who had AKI 3, only 6.5 were able to access dialysis. Of the 7 hospitals, only one of them has a hemodialysis center. In the other 6 hospitals, patients who needed dialysis were usually transferred. Considering that about 50% of patients with AKI 3 had hyperkalemia and/or metabolic acidosis, it is reasonable to assume that dialysis could reduce the number of deaths in ICU. In Western countries where the use of hemodialysis is common, mortality rates of AKI in ICU is generally lower [12, 26–28]. However, some retrospective studies applied the RIFLE classification of AKI to assess outcomes in critically ill patients, and showed death rates similar to those found in present study, probably because they selected older patients who had multiple comorbidities including about 20 to 30% with a history of CKD [28, 29].

Respiratory distress and the decrease in peripheral oxygen saturation were independent predictors of death. These alterations are pathophysiological consequence of many medical and surgical conditions. There is an interaction between respiratory failure and AKI. AKI worsens respiratory distress by three mechanisms: systemic inflammatory response mediated by cytokines, the invasion of the renal parenchyma by neutrophils and macrophages, the oxidative stress and the hypervolemia [30]. In turn, respiratory distress aggravates AKI by two mechanisms: firstly the conditions underlying respiratory distress, may cause water retention by inappropriately stimulating ADH secretion; secondly the increase in intrathoracic pressure and resultant decreased venous return caused by the mechanical positive pressure ventilation impair kidney perfusion [30].

Our analysis of a cohort of 476 patients from 7 ICU in Kinshasa using the AKIN classification showed a significant increase in the risk for mortality in patients who developed AKI compared with patients who did not. The increased risk was found to be proportional to the stage of AKI. These results are consistent with previous western studies [25]. The increased mortality rate in AKI 1 and AKI 2 poses the problem of ICU in SSA. It is about the equipment (respirator), the availability of drugs and training of intensivists without forgetting the management of late referall patients, who very often spend too long in small centers or with traditional practitioners before being transferred to hospitals [7].

For patients who had AKI 3, only 6.5 were able to access dialysis. Of the 7 hospitals, only one of them has a hemodialysis center. In the other 6 hospitals, patients who needed dialysis were usually transferred. Considering that about 50% of patients with AKI 3 had hyperkalemia and/or metabolic acidosis, it is reasonable to assume that dialysis could reduce the number of deaths in ICU. In Western countries where the use of hemodialysis is common, mortality rates of AKI in ICU is generally lower [12, 26–28]. However, some retrospective studies applied the RIFLE classification of AKI to assess outcomes in critically ill patients, and showed death rates similar to those found in present study, probably because they selected older patients who had multiple comorbidities including about 20 to 30% with a history of CKD [28, 29].

Respiratory distress and the decrease in peripheral oxygen saturation were independent predictors of death. These alterations are pathophysiological consequence of many medical and surgical conditions. There is an interaction between respiratory failure and AKI. AKI worsens respiratory distress by three mechanisms: systemic inflammatory response mediated by cytokines, the invasion of the renal parenchyma by neutrophils and macrophages, the oxidative stress and the hypervolemia [29]. In turn, respiratory distress aggravates AKI by two mechanisms: firstly the conditions underlying respiratory distress, may cause water retention by inappropriately stimulating ADH secretion; secondly the increase in intrathoracic pressure and resultant decreased venous return caused by the mechanical positive pressure ventilation impair kidney perfusion [30].

### Limitations

Our study has some limitations that must be considered in interpreting the results. Indeed, the size of the sample was not very large, a baseline reference creatinine (before hospitalization) was not available in many patients and the determination of serum creatinine was not calibrated. Furthermore we were unable to assess the long term survival of patients. The study has the merit of being a prospective cohort, and benefited from the participation of nephrologists, intensivists general practitioners and nurses of ICU who were trained on the screening of AKI.

## Conclusion

AKI is a prevalent problem in ICU and it affects short-term survival of critically ill patients, multiplying by six the risk of dying versus patients without AKI, especially during the first 3 days of hospitalization. Very few patients can get access to dialysis in the context of SSA. To combat it, we must focus on prevention and early treatment. For the most severe cases, it is urgent to promote dialysis in ICU and make it widely accessible.

## Abbreviations

ADH: Antidiuretic hormone
AKI: Acute kidney injury
AKIN: Acute kidney injury network
CKD: Chronic kidney disease
HIV: Human immunodeficiency virus
ICU: Intensive care unit
NSAID: Non-steroidal anti-inflammatory drugs
SSA: Sub Saharan Africa
